# Decreased CD8^+ ^T cell response to Epstein-Barr virus infected B cells in multiple sclerosis is not due to decreased HLA class I expression on B cells or monocytes

**DOI:** 10.1186/1471-2377-11-95

**Published:** 2011-08-03

**Authors:** Michael P Pender, Peter A Csurhes, Casey MM Pfluger, Scott R Burrows

**Affiliations:** 1 The University of Queensland, School of Medicine, Health Sciences Building, Royal Brisbane and Women's Hospital, Queensland 4029, Australia; 2 Department of Neurology, Royal Brisbane and Women's Hospital, Queensland 4029, Australia; 3 The University of Queensland Centre for Clinical Research, Royal Brisbane and Women's Hospital, Queensland 4029, Australia; 4 Cellular Immunology Laboratory, Queensland Institute of Medical Research, 300 Herston Rd., Brisbane 4029, Australia

## Abstract

**Background:**

Patients with multiple sclerosis (MS) have a decreased frequency of CD8^+ ^T cells reactive to their own Epstein-Barr virus (EBV) infected B cells. We have proposed that this might predispose to the development of MS by allowing EBV-infected autoreactive B cells to accumulate in the central nervous system. The decreased CD8^+ ^T cell response to EBV results from a general CD8^+ ^T cell deficiency and also a decreased proportion of EBV-specific T cells within the total CD8^+ ^T cell population. Because decreased HLA class I expression on monocytes and B cells has been reported in MS and could influence the generation and effector function of EBV-specific CD8^+ ^T cells, the present study was undertaken to measure the expression of HLA molecules on B cells and monocytes in patients with MS.

**Methods:**

We used flow cytometry to determine the proportions of T cells, natural killer cells, B cells and monocytes in peripheral blood mononuclear cells (PBMC) and to quantify the expression of HLA molecules on T cells, B cells and monocytes of 59 healthy subjects and 62 patients with MS who had not received corticosteroids or immunomodulatory therapy in the previous 3 months.

**Results:**

The levels of HLA class I and class II molecules expressed on T cells, B cells and monocytes were normal in patients with MS, with the exception of two patients with secondary progressive MS with very low class II expression on B cells. In confirmation of previous studies we also found that the percentage of CD8^+ ^T cells was significantly decreased whereas the percentage of CD4^+ ^T cells and the CD4:CD8 ratio were significantly increased in patients with MS compared to healthy subjects.

**Conclusions:**

The decreased CD8^+ ^T cell response to EBV-infected B cells in MS patients is not due to decreased HLA class I expression on monocytes or B cells. In a small proportion of patients decreased HLA class II expression on B cells might impair the CD8^+ ^T cell response to EBV by reducing CD4^+ ^T cell help.

## Background

A large body of evidence indicates that infection with the Epstein-Barr virus (EBV) has a role in the pathogenesis of multiple sclerosis (MS) [1-3]. Several hypotheses have been proposed to explain the role of EBV in the development of MS, including immunological crossreactivity between EBV and central nervous system (CNS) antigens [4], autoimmunity to αB-crystallin [5], EBV infection of autoreactive B cells [6], bystander damage from immune attack against EBV in the CNS [7], and interactions with other infectious agents such as human endogenous retroviruses [8]. It is important to determine the role of EBV in the pathogenesis of MS because of the potential to prevent and treat MS by controlling EBV infection, such as by vaccination against EBV [9]. In particular it is necessary to understand the exact relationship between EBV and the immune system in MS in order to ensure that these interventions are beneficial, not harmful.

EBV infection is normally kept under tight control by EBV-specific immune responses, especially by cytotoxic CD8^+ ^T cells which eliminate proliferating and lytically infected B cells [10]. We have hypothesized that a genetically determined defect in the elimination of EBV-infected B cells by cytotoxic CD8^+ ^T cells might predispose to the development of MS by allowing EBV-infected autoreactive B cells to accumulate in the central nervous system [3,6]. Studies of CD8^+ ^T cell reactivity to EBV using selected synthetic EBV peptides to stimulate the T cells have produced conflicting results, with reports of normal reactivity [11] and increased reactivity [12] in clinically isolated syndromes, and normal reactivity [12,13] and increased reactivity [14] in established MS. Studies using selected EBV peptides to assess T cell reactivity are limited by the fact that they do not provide a measure of the total T cell response to EBV, which encodes many different proteins. To overcome this it is necessary to measure the T cell response to autologous EBV-infected B cells. This provides a direct measure of the aggregate T cell response to EBV-infected B cells in each subject because it uses each person's natural antigen-processing mechanisms to present viral antigens at normal physiological concentrations on the surface of their own EBV-infected B cells and it represents the total T cell response to all EBV antigens presented by all HLA molecules on infected B cells in each subject [15]. Using this approach we have shown that patients with MS have a decreased frequency of CD8^+ ^T cells reactive to their own EBV-infected B cell lymphoblastoid cell lines (LCL) [15]. This results from a general CD8^+ ^T cell deficiency and also a decreased proportion of EBV-specific T cells within the total CD8^+ ^T cell population [16]. Our finding of decreased CD8^+ ^T cell reactivity to LCL in patients with MS [15] differs from a previous small study on 11 patients which reported a non-significant increase in the frequency of LCL-specific CD8^+ ^T cells [17], but is consistent with an early report of decreased T cell control of LCL outgrowth [18] and a recent report of a trend (p = 0.07) towards a decreased CD8+ T cell response to LCL [19].

In view of a previous report of decreased HLA class I expression on monocytes and B cells in patients with MS [20], which could impair the generation and effector function of EBV-specific CD8^+ ^T cells, we have undertaken the present study to quantify the level of HLA expression on B cells and monocytes in MS patients and healthy subjects.

## Methods

### Patients and controls

Blood was collected from 59 healthy subjects and 62 MS patients following informed consent. This study was approved by the Royal Brisbane & Women's Hospital Human Research Ethics Committee and The University of Queensland Medical Research Ethics Committee. All patients met the 2005 Revised McDonald Criteria for a diagnosis of MS [21]. The clinical course was relapsing-remitting (RRMS) in 23 patients, secondary progressive (SPMS) in 25 and primary progressive (PPMS) in 14. The patients had not received corticosteroids or immunomodulatory therapy for at least 3 months prior to venesection. Only two patients had ever received immunosuppressive drugs and these had been ceased four years before blood collection. An additional 7 patients had received interferon-β which had been ceased 6 months to 6 years before blood collection. Disability was assessed using the Kurtzke Expanded Disability Status Scale (EDSS) [22], and the MS Severity Score (MSSS) was determined from the EDSS and disease duration [23]. The demographic and clinical details of the healthy subjects and patients with MS are presented in Table 1.

**Table 1:** Characteristics of healthy subjects and patients with MS

	HC (*n *= 59)	All MS (*n *= 62)	RRMS (*n *= 23)	SPMS (*n *= 25)	PPMS (*n *= 14)
Age (years)	40.4 ± 10.8	45.5 ± 12.1	38.0 ± 9.7	50.8 ± 10.1	48.2 ± 13.3
Sex (% female)	67.8	69.3	73.9	68.0	64.3
Age of onset of MS (years)		33.5 ± 9.6	31.4 ± 10.6	33.4 ± 8.3	36.9 ± 9.7
Duration of MS (years)		12.8 ± 8.4	7.6 ± 6.5	18.0 ± 7.7	11.9 ± 7.1
EDSS score		5.1 ± 2.3	3.1 ± 1.8	6.3 ± 1.9	6.1 ± 1.6
MSSS		6.2 ± 2.6	4.9 ± 2.8	6.9 ± 2.4	7.4 ± 1.9

All values except for sex presented as mean ± standard deviation.

HC: healthy control subjects, All MS: total group of MS patients, RRMS: relapsing-remitting MS, SPMS: secondary progressive MS, PPMS: primary progressive MS, EDSS: Expanded Disability Status Scale, MSSS: MS Severity Score.

### Flow cytometry

Peripheral blood mononuclear cells (PBMC) were separated by density centrifugation and cryopreserved, as previously described [15]. PBMC samples were thawed and cultured for 24 h to allow cells to rest and re-express cell surface receptors. Propidium iodide staining demonstrated > 99% viability within the lymphocyte and monocyte forward scatter/side scatter gate for each sample used. PBMC samples were assessed using a Becton Dickinson FACSCalibur flow cytometer to determine the percentages of CD3^+ ^T cells, CD4^+^CD3^+ ^T cells, CD8^+^CD3^+ ^T cells, CD16^+^CD3^-^/CD56^+^CD3^- ^natural killer (NK) cells, CD19^+ ^B cells and CD14^+ ^monocytes within the combined lymphocyte and monocyte gates. To quantify expression of HLA class I or class II molecules, PBMC were co-stained with either anti-HLA-ABC or anti-HLA-DR/DP/DQ in combination with anti-CD3 (T cells), anti-CD19 (B cells) or anti-CD14 (monocyte) antibodies. HLA-DR expression was also measured in 30 of the healthy subjects and 33 of the MS patients. Calibrite beads (BD Pharmingen) and FACSComp software were used daily to ensure calibrated, comparable measurement of geometric mean fluorescence intensity (MFI). Antibodies were directly conjugated to allophycocyanin (anti-CD3, anti-CD19 and anti-CD14), R-phycoerythrin (anti-CD4, anti-CD8, anti-CD16 and anti-CD56) or fluorescein isothiocyanate (anti-HLA-ABC, anti-HLA-DR/DP/DQ and anti-HLA-DR) (BD Pharmingen, San Diego, California, USA). CellQuest software (BD Biosciences) was used for acquisition and analysis of flow cytometry data.

### Data analysis and statistics

Statistical analyses were performed using GraphPad Prism version 5.04 (Graphpad Software Inc., San Diego, California, USA, http://www.graphpad.com). For comparisons between the whole group of MS patients and healthy subjects, Student's *t *test or the Mann-Whitney rank sum test was used, according to the distribution of the data. For comparison between each of the subtypes of MS (RRMS, SPMS and PPMS) and healthy subjects, one-way analysis of variance with Dunnett's test for multiple comparisons was used. The CD4:CD8 ratios were log transformed to make the distributions approximately normal prior to analysis. Differences were considered significant for *p *< 0.05.

## Results

Table 2 shows the proportions of lymphocytes and monocytes in the peripheral blood. The percentages of CD3^+ ^T cells, NK cells, B cells and monocytes were normal in the total group of MS patients, as well as in the RRMS, SPMS and PPMS subgroups, with the exception of an increased percentage of CD3^+ ^T cells in PPMS. The percentage of CD8^+ ^T cells was significantly decreased whereas the percentage of CD4^+ ^T cells and the CD4:CD8 ratio were significantly increased in the total group of MS patients compared to healthy subjects; these changes were more pronounced in SPMS and PPMS than in RRMS. The levels of expression of HLA class I molecules on T cells, B cells and monocytes were normal in the total group of MS patients and in the RRMS, SPMS and PPMS subgroups (Table 2). Analysis of HLA class I expression with the W6/32 antibody used by Li and colleagues [20] also demonstrated normal HLA class I expression on lymphocytes and monocytes in the MS patients (data not shown). The levels of HLA class II expression on T cells, B cells and monocytes were also normal in the total group of MS patients, as well as in the RRMS, SPMS and PPMS subgroups (Table 2), with the exception of very low levels of B cell expression (MFI = 1077 and 1139) in 2 patients with SPMS. HLA-DR expression on B cells was also assessed in one of these 2 patients and found to be very low (MFI = 2404). HLA-DR expression on B cells and monocytes was otherwise normal in the MS patients tested (Table 3).

**Table 2:** PBMC subsets and HLA class I and class II expression

	HC (*n *= 59)	All MS (*n *= 62)	RRMS (*n *= 23)	SPMS (*n *= 25)	PPMS (*n *= 14)
% CD3^+ ^T cells	72.8 ± 0.8	74.4 ± 1.0	74.2 ± 1.5	72.2 ± 1.7	78.9 ± 1.5
*p *value		0.216*			< 0.05
% CD4^+^CD3^+ ^T cells	46.2 ± 1.1	52.2 ± 1.2	49.7 ± 1.9	51.1 ± 1.8	58.1 ± 1.9
*p *value		< 0.001*		< 0.05	< 0.001
% CD8^+^CD3^+ ^T cells	23.1 ± 0.8	19.8 ± 0.7	21.7 ± 1.1	18.8 ± 1.0	18.3 ± 1.6
*p *value		0.007†		< 0.01	< 0.05
CD4:CD8 ratio	2.2 ± 0.1	3.0 ± 0.2	2.5 ± 0.2	3.0 ± 0.3	3.7 ± 0.5
*p *value		< 0.001†		< 0.01	< 0.001
% NK cells	8.1 ± 0.6	8.1 ± 0.6	9.1 ± 1.1	8.0 ± 1.1	6.5 ± 1.0
*p *value		0.553†			
% B cells	13.1 ± 0.6	11.7 ± 0.7	11.1 ± 1.1	13.0 ± 1.1	10.5 ± 1.1
*p *value		0.143*			
% monocytes	7.7 ± 0.6	7.6 ± 0.6	8.2 ± 1.1	8.1 ± 1.0	5.4 ± 1.0
*p *value		0.752†			
T cell HLA class I MFI	2015 ± 60	2072 ± 46	1964 ± 61	2082 ± 73	2223 ± 107
*p *value		0.445*			
T cell HLA class II MFI	14.0 ± 0.5	13.7 ± 0.6	13.3 ± 0.8	13.3 ± 0.7	15.0 ± 1.7
*p *value		0.406†			
B cell HLA class I MFI	3838 ± 132	4063 ± 123	4037 ± 231	3919 ± 164	4362 ± 261
*p *value		0.214*			
B cell HLA class II MFI	4384 ± 146	4273 ± 153	4313 ± 210	3951 ± 265	4784 ± 315
*p *value		0.605*			
Monocyte HLA class I MFI	4948 ± 221	4941 ± 189	4823 ± 282	4837 ± 355	5322 ± 297
*p *value		0.982*			
Monocyte HLA class II MFI	2587 ± 218	2907 ± 236	3053 ± 418	2578 ± 317	3254 ± 561
*p *value		0.474†			

All values presented as mean ± standard error.

PBMC: peripheral blood mononuclear cells, HC: healthy control subjects, All MS: total group of MS patients, RRMS: relapsing-remitting MS, SPMS: secondary progressive MS, PPMS: primary progressive MS, NK: natural killer, MFI: mean fluorescence intensity.

All *p *values are for comparisons with HC, using either Student's *t *test* or the Mann-Whitney rank sum test† for the comparison with All MS, and using one-way analysis of variance with Dunnett's test for multiple comparisons for comparisons with RRMS, SPMS and PPMS; *p *values < 0.05 are considered significant. In the right three columns of the table, *p *values are corrected for multiple comparisons using Dunnett's test and are > 0.05 unless otherwise indicated. The CD4:CD8 ratios have been log transformed to make the distributions approximately normal prior to analysis.

**Table 3:** HLA-DR expression

	HC (*n *= 30)	All MS (*n *= 33)	RRMS (*n *= 10)	SPMS (*n *= 15)	PPMS (*n *= 8)
B cell HLA-DR MFI	4745 ± 127	4729 ± 147	4894 ± 209	4489 ± 279	4974 ± 135
*p *value		0.940	> 0.05	> 0.05	> 0.05
Monocyte HLA-DR MFI	4122 ± 337	4321 ± 314	4657 ± 683	4122 ± 384	4273 ± 729
*p *value		0.655	> 0.05	> 0.05	> 0.05

All values presented as mean ± standard error.

HC: healthy control subjects; All MS: total group of MS patients, RRMS: relapsing-remitting MS, SPMS: secondary progressive MS, PPMS: primary progressive MS, MFI: mean fluorescence intensity.

All *p *values are for comparisons with HC, using the Mann-Whitney rank sum test for the comparison with All MS, and using one-way analysis of variance with Dunnett's test for multiple comparisons for comparisons with RRMS, SPMS and PPMS; *p *values < 0.05 are considered significant.

## Discussion

In the present study we have found that patients with MS have normal expression of HLA class I molecules on T cells, B cells and monocytes. Our findings stand in contrast to a previous study reporting low levels of HLA class I expression on T cells, B cells and monocytes in MS patients [20], but are consistent with a report of normal HLA class I expression on monocytes in MS [24] and with our previous finding of normal HLA class I expression on EBV-infected LCL in MS patients [15]. We therefore conclude that the decreased CD8^+ ^T cell response to EBV-infected B cells in MS patients [15,16] is not due to decreased HLA class I expression on monocytes or B cells.

We also found normal expression of HLA class II molecules on T cells, B cells and monocytes in patients with MS, with the exception of two patients with SPMS who had very low class II expression on B cells. Low B cell HLA class II expression might impair the CD8^+ ^T cell response to EBV by reducing CD4^+ ^T cell help, which normally stimulates the expansion of EBV-specific CD8^+ ^T cells by producing interleukin-2 [25]. Our finding of normal HLA-DR expression on monocytes in MS is consistent with the report of Kouwenhoven and colleagues [24].

Our findings of an increased proportion of CD4^+ ^T cells, a decreased proportion of CD8^+ ^T cells and an increased CD4:CD8 ratio in the blood of patients with MS are consistent with previous studies [18,26-33]. Our observation that CD8^+ ^T cell deficiency is more marked in SPMS and PPMS than in RRMS corroborates previous studies reporting that the decrease in CD8^+ ^T cells is most pronounced in progressive MS [29-31]. We did not measure the absolute counts of CD4^+ ^T cells and CD8^+ ^T cells but previous studies have shown that the absolute number of CD4^+ ^T cells is normal and the absolute number of CD8^+ ^T cells is decreased in MS [30,32,33]. Future studies should be directed towards determining the cause of the CD8^+ ^T cell deficiency, which we propose is genetically determined. The CD4/CD8 T cell ratio is genetically controlled [34], with at least some of the responsible genes being located in the HLA complex [35]. CD8^+ ^T cell deficiency is a feature of many chronic autoimmune diseases and is also found in healthy blood relatives of patients with autoimmune diseases [36-38], indicating that it is genetically determined and not secondary to the disease process.

## Conclusions

The decreased CD8^+ ^T cell response to EBV-infected B cells in MS patients is not due to decreased HLA class I expression on monocytes or B cells. In a small proportion of patients decreased HLA class II expression on B cells might impair the CD8^+ ^T cell response to EBV by reducing CD4^+ ^T cell help.

## Competing interests

The authors declare that they have no competing interests.

## Authors' contributions

MPP planned the study, recruited and assessed the patients, helped analyze the results, and wrote the manuscript. PAC performed the laboratory studies and analyzed the data. CMMP assisted with the laboratory studies. SRB helped plan the study and analyze the data. All authors read and approved the final manuscript.

## Pre-publication history

The pre-publication history for this paper can be accessed here:

http://www.biomedcentral.com/1471-2377/11/95/prepub
